# Program 25th Annual Meeting Mississippi Valley Medical Association, Chicago, October 3–6, 1999

**Published:** 1899-09

**Authors:** 


### Society Notes.

### Program 25th Annual Meeting Mississippi Valley Medical Association, Chicago, October 3–6, 1999.

OFFICERS.

President.............................Duncan Eve, Nashville.
First Vice-President....................A. J. Ochsner, Chicago.
Second Vice-President...................J. C. Morfit, St. Louis.
Secretary.........................Henry E. Tuley, Louisville.

Treasurer......................Dudley S. Reynolds, Louisville.
Chairman Committee Arrangements........H. N. Moyer, Chicago.
Address in Medicine: Dr. J. A. Witherspoon, Nashville.
Address in Surgery: Dr. Lewis S. McMurtry, Louisville.

MEDICAL SECTION.

1. Enzymes and Immunity.......Chas. T. McClintock, Detroit.
2. Recent Physio-Chemic Researches as to the Physiologic Action of Lecithin and other Organic Phosphorus Compounds.......L. H. Warner, Brooklyn, N. Y.
3. Communal Hygiene.....Ernest B. Sangree, Nashville, Tenn.
4. Some Phases of Malaria—Quinine In........................Wm. Britt Burns, Deckerville, Ark.
5. The Treatment of Pulmonary Tuberculosis by Inhalation of Antiseptic Nebulæ.......................................Homer M. Thomas, Chicago, Ill.
6. The Management of Cases of Pulmonary Phthisis at Health Resorts..........Chas. F. McGahan, Aiken, S. C.
7. The Treatment of Acute Lobar Pneumonia.............................Ramon D. Garcin, Richmond, Va.
8. The Art of Diagnosis......E. L. Larkins, Terre Haute, Ind.
9. Do We Need to Think?.....................................Wm. O'Neall Mendenhall, Richmond, Ind.
10. The Evils: Their Causes, and the Remedy that will Edify Medicine in the United States...................................A. M. Osness, Dayton, Ohio.
11. Two Cases of Typhoid Fever with Unusual Complications, in Very Young Children...................................E. B Montgomery, Quincy, Ill.
12. Further Observations on the Treatment of the Abdominal Viscera through the Colon...................................Fenton B. Turck, Chicago, Ill.
13. Report of a Case of Complete Hernia of the Pregnant Uterus.............M. V. Anderson, Toledo, Ohio.
14. Leptomeningitis...Frank Parsons Norbury, Jacksonville, Ill.
15. Pathogenesis of Functional Nerve Diseases and its Prophylactic Indications..John Punton, Kansas City, Mo.
16. The Association of Hysteria with Organic Disease of the Nervous System..Philip F. Zenner, Cincinnati, Ohio.
17. The Clinical Psychiatrist in General Practice...........................C. H. Hughes, St. Louis, Mo.

18. Temperament and its Influence....................................Albert E. Sterne, Indianapolis, Ind.
19. Obstipation and its Radical Treatment...........................Thomas Charles Martin, Cleveland, Ohio.
20. Intestinal Auto-Intoxication, its Prevention and Treatment............Wm. F. Barclay, Pittsburgh, Pa.
21. Indigestion in Infants and Children...............................James H. Taylor, Indianapolis, Ind.
22. Lithiasis................R. Alexander Bate, Louisville, Ky.
23. Nephrolithiasis............A. H. Cordier, Kansas City, Mo.
24. The Therapeutics of Infectious Conjunctivitis.........................Dudley S. Reynolds, Louisville, Ky.
25. Supra-renal Gland as a Therapeutic Agent in Ophthalmology, Otology and Rhinology.............................Flavel B. Tiffany, Kansas City, Mo.
26. Introversion of the Iris.......L. W. Beardsley, St. Louis, Mo.
27. The Successful Treatment of a Case of Graves' Disease as an Auto-Intoxication.......................................Chas. L. Minor, Asheville, N. C.
28. The Treatment of Dysentery.......................................J. W. Knowlton, Paint Rock, Ala.
29. A Contribution to the Study of Lung Reflexes.............................Marion K. Bowles, Joliet, Ill.

SURGICAL SECTION.

30. Vesico-Rectal Anastomosis..........J. Frank, Chicago, Ill.
31. Intolerant Ulceration of the Rectum, with the Report of Five Cases........Sterling B. Taylor, Columbus, Ohio.
32. Modern Surgical Treatment of Hemorrhoids,...............................G. M. Blech, Chicago, Ill.
33. An Arm Saved after being Run Over by a Railway Locomotive and Crushed. .S. L. Kilmer, South Bend, Ind.
34. Urethral Endoscopy...........W. R. Blue, Louisville, Ky.
35. The Value of Prostatic Examination...............................J. Leland Boogher, St. Louis, Mo.
36. The Technique of Abdominal Incisions, Peritoneal and Extra-Peritoneal......S. E. Milliken, Dallas, Texas.
37. Mammoth Ovarian Cysts: Report of a Tumor weighing 245 Pounds.....Jas. B. Bullitt, Louisville, Ky.
38. Some Causes of Death after Abdominal Section.............................Louis Frank, Louisville, Ky.

39. Intestinal Obstruction from Gall Stones..........
.....................J. Wesley Boveé, Washington, D. C.
40. Obstructive Growths of the Pylorus, with Report of a Successful Case of Pylorectomy..............
..........................J. E. Allaben, Rockford, Ill.
41. What Becomes of the Medicinally Treated Cases of Appendicitis?........Lewis Schooler, Des Moines, Iowa.
42. Appendicitis from a Medical Standpoint..........
...........................I. N. Love, St. Louis, Mo.
43. A Plea for Early Operation in Appendicitis........
......................A. M. Hayden, Evansville, Ind.
44. Surgical Features of Appendicitis...............
........................Hal C. Wyman, Detroit, Mich.
45. A Study of Twenty-seven Cases of Appendicitis....
................Frank T. Merriwether, Asheville, N. C.
46. Surgery of the Turbinated Bones................
........................J. A. Stucky, Lexington, Ky.
47. Nasal Stenosis Due to Defective Septa, and its Treatment with or without Thickening of the Convex Side......................John J. Kyle, Marion, Ind.
48. Mastoid Operation, with Report of Cases..........
......................Geo. F. Keiper, Lafayette, Ind.
49. Beta-eucaine as an Anesthetic in Eye Surgery......
..........................W. H. Poole, Detroit, Mich.
50. The Surgical Treatment of Chronic Frontal Sinusitis..............Richmond McKinney, Memphis, Tenn.
51. Observations on Surgery of the Brain, Based on Clinical and Experimental Evidence.............
.....................George W. Crile, Cleveland, Ohio.
52. Removal of the Cervical Sympathetic for Epilepsy, Exophthalmic Goitre, and Glaucoma..........
.....................Emory Lanphear, St. Louis, Mo.
53. Treatment of Certain Ocular Diseases by Excision of the Cervical Sympathetic Ganglia...........
....................James Moores Ball, St. Louis, Mo.
54. Suture Materials in Surgery.....Jos. Price, Philadelphia, Pa.
55. The General Treatment of Patients Before, During, and After Surgical Operations..............
.......................Fenton B. Turck, Chicago, Ill.
56. The Modern Small Bore Projectile...............
......................Aug. Schachner, Louisville, Ky.

57. The Effects of the Automatic Mauser Pistol........
........................J. D. Griffith, Kansas City, Mo.
58. Surgical Tolerance and Results..................
.........................F. F. Bryan, Georgetown, Ky.
59. The Treatment of Gonorrhea in the Female........
.........................A. Ravogli, Cincinnati, Ohio.
60. Inflammation of the Verumontanum......Indianapolis, Ind.

